# Steroidogenic factor-1 (NR5A1): orphan nuclear receptor finds a home in human reproduction, and beyond

**DOI:** 10.1016/j.ebiom.2024.104984

**Published:** 2024-01-28

**Authors:** John C. Achermann

**Affiliations:** Genetics and Genomic Medicine Research and Teaching Department, UCL Great Ormond Street Institute of Child Health, University College London, London WC1N 1EH, United Kingdom

The quest to discover a “master-regulator” of adrenal and reproductive function began in the late 1980s, when it became apparent that specific transcription factors often played key roles in the development and function of different organs. Subsequently, the research groups of Keith Parker and Ken-ichirou Morohashi independently cloned a nuclear receptor transcription factor that could have this master-regulator role, which was termed steroidogenic factor-1 (SF-1, also known as Ad4BP/FTZ-F1 and officially as *NR5A1*/NR5A1).^1^

SF-1 is an “orphan” nuclear receptor (with no high-affinity ligand) that promotes the transcription of an array of different genes involved in endocrine development, steroidogenesis and metabolism. The generation of a knockout mouse model of *Nr5a1* in 1994, which had impaired adrenal and gonad development and brain ventromedial hypothalamic (VMH) anomalies, soon provided conclusive proof that SF-1 was indeed an essential player in endocrine biology.^2^

The next goal was to establish whether biologically important variants in SF-1/*NR5A1* occurred in humans. Initial efforts understandably focussed on individuals with a similar phenotype to the mouse model; namely, primary adrenal insufficiency and complete gonadal (testicular) dysgenesis. This combination of clinical features is very uncommon, but a heterozygous change in a critical nuclear receptor DNA-binding motif (“P-box”) was first reported 25 years ago in 1999.^3^ Although occasional cases of adrenal insufficiency linked to disruptive variants in SF-1/*NR5A1* were subsequently identified, it seemed likely that SF-1-associated conditions would ultimately be confined to the category of “biologically interesting, but very rare”.

This assumption changed following the analysis of SF-1/*NR5A1* in individuals with a wider range of features. Although primary adrenal insufficiency associated with variants in SF-1/*NR5A1* was not common, human gonad (and especially testis) development and function seemed much more sensitive to the effects of reduced SF-1 function. Heterozygous variants were reported in children and adults with a wide spectrum of phenotypes ranging from testicular dysgenesis and severe hypospadias to male infertility in 46,XY individuals, and primary ovarian insufficiency (POI) in 46,XX individuals.^4^, ^5^, ^6^ Specific variants in a hot spot of SF-1 (p.Arg92) were even shown by several groups to cause the ovary to develop testicular tissue (ovotestis).

In the January 2024 issue of eBioMedicine, Kouri and colleagues^7^ report findings of the “SF1Next” study. This work presents collaborative clinical and genetic data on SF-1/*NR5A1* on a global scale. The authors had planned to include 100 individuals, but ended up with almost 200. The authors have been able to assimilate a large body of clinical and molecular data from those with potentially pathogenic SF-1/*NR5A1* variants, including extended data on previously reported cases. The demographic focus was mostly on children and young people with differences in sex development (DSD), rather than on adults with impaired spermatogenesis or POI.

So, what does SF1Next add? From a clinical perspective, this study shows the benefits of bringing together large international cohorts in the field of “rare diseases”. For example, a previously reported potential association between SF-1 disruption and hyposplenia/asplenia has emerged much more strongly,^8^ the consequences of which will need to be considered in clinical practice. Associated adrenal insufficiency remains rare, although this was not studied systematically. A potential role of SF-1 in obesity, that was reported following VMH disruption in the mouse, does not appear to be obviously borne out in humans, although long-term follow up data are needed. This is all important clinical information to know.

From a genetic perspective, the authors show a potential role for digenic or oligogenic influences: that is, variants in additional genes that may combine with disruption of SF-1/*NR5A1* and potentially influence phenotypic penetrance. Oligogenic influences have been proposed in several endocrine conditions, such as hypogonadotropic hypogonadism or primary ovarian insufficiency.^9^ Unfortunately, a systematic approach to extensive sequencing or resequencing of all individuals in SF1next could not be undertaken, and the number of individuals with clearly impactful second changes was modest, so obtaining greater certainty for the exact role of oligogenic genetic effects is needed. Nevertheless, this work adds to the concept that multiple genetic events may be important, especially in complex developmental networks or in situations where phenotypic penetrance is variable.

Finally, from a developmental biology perspective, SF1Next really highlights the major role that SF-1/*NR5A1* plays in human reproductive development, function and related conditions. Along with the androgen receptor (AR/*NR3C4*) and DAX-1/*NR0B1*, SF-1/*NR5A1* is one of the most common nuclear receptor-related conditions in humans, with a large number of different variants and wide spectrum of clinical features emerging.^10^

This work also highlights future challenges and questions. Long-term follow up will be needed to establish: if there is any risk of adrenal insufficiency with age, or under stress; what the gonadal tumour risk might be; how likely ovarian dysfunction is with time in 46,XX individuals who harbour SF-1/*NR5A1* variants; and whether assisted reproductive strategies have a role. Genetic counselling can be difficult, especially in the context of variable penetrance and – now – potential oligogenic effects in some situations. The American College of Medical Genetics and Genomics (ACMG) criteria for defining pathogenicity can be challenging in these contexts. This may be particularly true when the effects of SF-1 disruption may be “locked” in a dosage sensitive developmental time window, or influenced by other hard-to-define mechanisms such as skewed allelic expression.

Despite these challenges, SF1Next clearly serves to further establish SF-1-associated conditions as a major entity, 25 years after the first report of a pathogenic variant in humans. We look forward to what the (SF1) next 25 years of clinical and basic research will bring.

## Contributors

Conceptualisation–JCA; Writing–JCA. JCA is the sole author of the commentary.

## Declaration of interests

JCA has no conflicts of interest to disclose.
